# Identifying ionic interactions within a membrane using BLaTM, a genetic tool to measure homo- and heterotypic transmembrane helix-helix interactions

**DOI:** 10.1038/srep43476

**Published:** 2017-03-07

**Authors:** Christoph Schanzenbach, Fabian C. Schmidt, Patrick Breckner, Mark G. Teese, Dieter Langosch

**Affiliations:** 1 Munich Center For Integrated Protein Science (CIPSM) at the Lehrstuhl für Chemie der Biopolymere, Technische Universität München, Weihenstephaner Berg 3, 85354 Freising, Germany

## Abstract

The assembly of integral membrane protein complexes is frequently supported by transmembrane domain (TMD) interactions. Here, we present the BLaTM assay that measures homotypic as well as heterotypic TMD-TMD interactions in a bacterial membrane. The system is based on complementation of β-lactamase fragments genetically fused to interacting TMDs, which confers ampicillin resistance to expressing cells. We validated BLaTM by showing that the assay faithfully reports known sequence-specific interactions of both types. In a practical application, we used BLaTM to screen a focussed combinatorial library for heterotypic interactions driven by electrostatic forces. The results reveal novel patterns of ionizable amino acids within the isolated TMD pairs. Those patterns indicate that formation of heterotypic TMD pairs is most efficiently supported by closely spaced ionizable residues of opposite charge. In addition, TMD heteromerization can apparently be driven by hydrogen bonding between basic or between acidic residues.

Most integral membrane proteins form homo- or heterotypic dimers or higher oligomers^1^. The assembly of such complexes frequently depends on interactions between TMDs, a topic which has received considerable attention over the past two decades. TMD-TMD interactions involve all kinds of physical forces known to stabilize proteins and their interactions; these include van der Waals forces, hydrogen bonds and aromatic interactions^2^,^3^,^4^,^5^. In addition, charge-charge interactions have previously been implied in TMD-TMD interactions^6^. Although the ionizable residues Asp, Glu, Lys, and Arg collectively account for only 6.6% of all residues within TMDs, a database analysis showed that 46% of the investigated TMDs contain at least one of these amino acids^7^. Further, polar residue types are strongly conserved, which indicates their structural and/or functional relevance^8^. Indeed, ionic TMD-TMD interactions support the oligomeric assembly of functionally diverse membrane protein complexes such as the T-cell receptor complex that is composed of single-span subunits. Assembly of the complete oligomer rests on interaction of one basic residue of the central αβ receptor with a pair of acidic residues within any of the CD3γε, CD3δε, and ζζ signalling homodimers^9^. Similarly, a single Asp residue within the TMD of the immuno signalling receptor DAP12 binds to a basic residue within the TMDs of its associated receptors^10^,^11^. Dynamic ionic TMD-TMD interactions are important for the gating of voltage-activated ion channels. There, sequential formation of ion pairs between Arg residues of the S4 TMD with acidic residues of different surrounding TMDs stabilizes S4 in the membrane and permits its voltage-triggered movement^12^,^13^.

Methods used to investigate TMD-TMD interactions include gel-shift assays, analytical ultracentrifugation, fluorescence resonance energy transfer, thiol disulfide interchange, and the steric trap^14^,^15^,^16^,^17^.

Over the last 20 years, genetic reporter assays of TMD interactions have also proven to be a powerful tool in the understanding of the residues, motifs, and forces involved in TMD interactions. These genetic tools convert the interaction of TMDs in a natural cell membrane into reporter gene expression. In particular, the ToxR-based assays in *E. coli*, which include ToxR^18^, TOXCAT^19^, as well as the recently developed dsTβL^20^ and TOXGREEN^21^ have provided insights into the TMD-driven homodimerization of single-span membrane proteins, where high-resolution structures are especially lacking. In these assays, TMDs of interest are fused to a cytoplasmic ToxR domain. Where TMD interaction brings the cytoplasmic ToxR domain in close proximity, the ToxR dimer acts as a transcriptional regulator which drives reporter gene expression thus giving a measure of TMD dimerization. The use of reporter genes encoding antibiotic resistance in ToxR assays has allowed mutational analyses of candidate TMD-TMD interfaces as well as the selection of self-interacting helices from partially randomized TMD sequence libraries. These analyses uncovered the importance of interfacial GxxxG^22^, QxxS^23^, and serine/threonine-containing^24^ motifs as well as aromatic^25^,^26^,^27^ and carboxamide^28^,^29^ residues. Library screens also produced more complex motifs where phenylalanine^30^, histidine/serine/threonine^31^ or ionizable residues^32^ combine with GxxxG motifs to form strong self-interaction.

Assays reporting heterotypic TMD-TMD interaction have been based on dominant-negative versions of ToxR-based assays. There, a heterotypic interaction between a TMD fused to ToxR and a TMD fused to a transcriptionally inactive ToxR’ mutant competes with homotypic ToxR-ToxR assembly, thus reducing reporter gene expression^31^,^33^. DN-AraTM is another dominant-negative assay based on AraC^34^. The ToxR system has been expanded to Multi-Tox that allows the study of multi-span protein transmembrane domain oligomerization^35^.

To understand heterotypic TMD interactions, including the role of oppositely charged residues for them, there is a clear need for a genetic reporter assay that combines the measurement of dimer affinity, acts as a selection tool, and only gives a response for membrane-integrated proteins. None of the dominant-negative heterotypic assays^31^,^33^,^34^ has yet been used in library screens. The GALLEX assay directly measures heterotypic TMD interaction by detecting downregulated β-galactosidase gene expression in response to the dimerization of TMDs fused to LexA DNA binding domains^36^. Since TMD heterodimerization causes repression of reporter gene synthesis, GALLEX is impractical for positive selection. A split ubiquitin-based method, termed mammalian-membrane two-hybrid assay (MaMTH), detects integral membrane protein-protein interactions in human cells via the induction of green fluorescent protein or luciferase expression^37^. To our knowledge, no screen of a combinatorial library has yet been performed with MaMTH. In the bacterial-two-hybrid (BACTH) system, heterotypic TMD interactions drive the reconstitution of adenylate cyclase from fragments. Adenylate cyclase produces cAMP which cooperates with *c*atabolite gene activator protein in driving β-galactosidase expression^38^,^39^. BACTH has recently been reconfigured to a system allowing for library screening. In this novel BACTH derivative, the β-lactamase reporter has been replaced by the *mal* regulon genes. In response to TMD-TMD interaction, maltose metabolizing enzymes accumulate in the cytoplasm of cells of an adenylate cyclase deficient *E. coli* strain, thus allowing their growth on minimal medium with maltose as the only carbon source^40^. Screening a combinatorial TMD library with this system has identified a diverse set of TMD pairs that exhibit an abundance of G,A,SxxxG,A,S motifs.

All these tools depend on the activity of transcription regulation domains extending from the membrane into the cytoplasm of a cell. Thus, it is unknown to which extent hybrid protein that is not correctly integrated into the membrane and aggregated in the cytoplasm may contribute to reporter gene expression. Signal generation from periplasmically localized domains responding to TMD-TMD interaction is therefore a highly desirable feature of a genetic tool.

Here, we present an assay which utilizes the powerful split β-lactamase system^41^ as the first TMD interaction assay in which the dimerization signal is derived from the periplasmic domain. As the system is based on antibiotic resistance, we use survival of expressing bacteria in ampicillin-containing media to isolate pairs of TMDs containing ionizable amino acids from a focussed combinatorial library. The isolated sequences provide novel insights into how patterns of ionizable amino acids support heterotypic TMD-TMD interactions.

## Results

### Development and validation of the BLaTM system

A protein complementation assay based on split β-lactamase had previously been used to monitor interactions between soluble proteins. Thereby, genetic fusion of β-lactamase fragments with interacting proteins had resulted in non-permanent reconstitution of enzyme activity *in vitro* and *in cellulo*^41^.

Here, we developed the BLaTM system which is based on split β-lactamase. In this system, two hybrid proteins are encoded by separate plasmids having similar copy numbers and are expressed under control of the arabinose promoter pBAD (Supplementary Fig. S1). We initially confirmed that co-expression of N-terminal (N-BLa) and C-terminal (C-BLa) β-lactamase fragments, both of which were genetically fused to the self-interacting soluble GCN4 leucine zipper^42^, in the *E. coli* periplasmic space successfully reconstitutes β-lactamase activity. Indeed, turnover of the β-lactamase substrate nitrocefin doubled after co-expression of GCN4_N-BLa and GCN4_C-BLa fragments, relative to their individual expression or non-expressing control cells (Supplementary Fig. S2a). In an alternative approach to measure the reconstitution of β-lactamase from these fragments, we determined their ability to confer antibiotic resistance to the bacterial cells. Indeed, the ampicillin concentration permitting the survival of 50% of cells in liquid media (the LD_50_ value as determined by measuring optical density) rose ~40-fold upon co-expressing both fragments (Supplementary Fig. S2b). Compared to the nitrocefin assay, the antibiotic survival assay has therefore a superior discriminating power. The workflow used to determine LD_50_ values is schematized in Supplementary Figure S3a.

**Figure 1:**
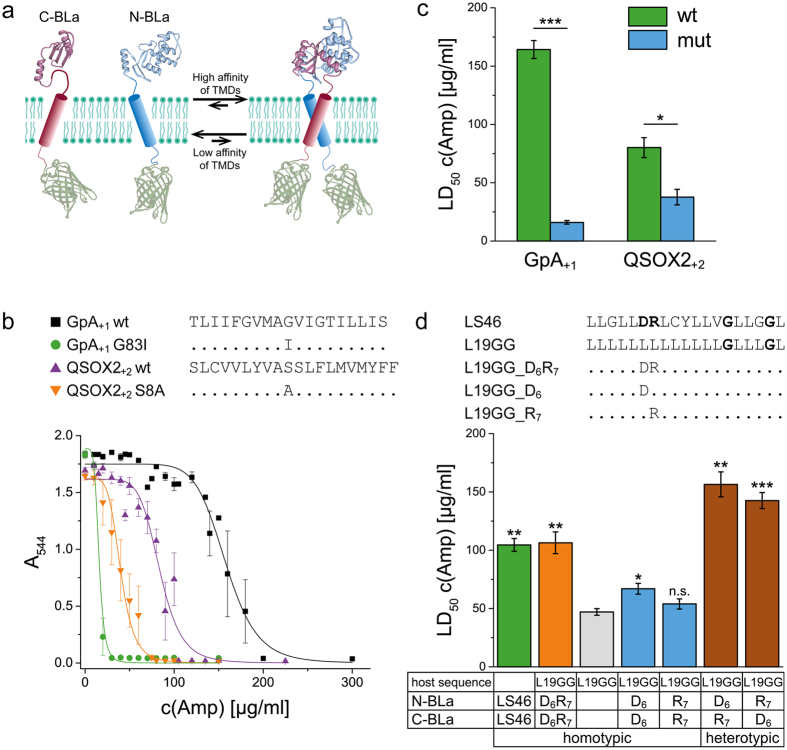
Design and validation of the BLaTM assay. (**a**) Scheme depicting the main features of the system. (**b**) Example of dose-response curves where the decrease of *E. coli* cell density (A_544_) to increasing ampicillin concentration is used to calculate LD_50_ values. (**c**) LD_50_ values derived from part. (**b**) These values characterize the homotypic interaction of the wild-type (wt) GpA and QSOX2 TMDs and the impact of point mutations (mut). TMDs were inserted into the hybrid proteins at orientations where wild-type/mutant LD_50_ ratios were maximal (see: Supplementary Fig. S4). (**d**) Sequence-specific homo- and heterotypic interaction driven by ionizable residues. The LS46 orientation used here leads to superior LD_50_ values compared to other orientations (not shown). All data were generated using BLaTM 1.1 in *E. coli* BL21 (**b**,**c**) or in *E. coli* JM83 cells (**d**) and represent means ± SEM, n = 4 separate transformations. The denomination D_6_R_7_ corresponds to D_5_R_6_ previously used^32^. Single, double, or triple asterisks denote statistical significance at the 0.05, 0.01, or 0.001 confidence levels (relative to the mutants in part (**c**) or to L19GG in (**d**)).

In order to measure the homo- or heterotypic interaction between candidate TMDs or to isolate high-affinity TMDs from a combinatorial library, we designed a pair of hybrid proteins into which a given TMD sequence can be inserted at the genetic level. An inserted TMD is connected via a flexible linker to the C-terminus of either N-BLa or C-BLa and via a rigid linker to superfolder green fluorescent protein (GFP) for ease of protein detection. N-terminal pelB (BLaTM version 1.1) or ompA (BLaTM version 1.2) cleavable signal peptides direct either protein to the inner bacterial membrane and determine their N_out_ topology (Fig. 1a and Supplementary Fig. S1). The efficiency of TMD-TMD interaction within the inner bacterial membrane is thus reflected by the activity of reconstituted β-lactamase residing in the periplasmic space.

We first validated BLaTM 1.1 using two TMDs known to homodimerize: the high-affinity TMD of glycophorin A (GpA^18^,^19^) and the medium-affinity TMD of quiescin sulfhydryl oxidase 2 (QSOX2^43^). Indeed, we found that the LD_50_ conferred by the GpA TMD exceeds that of the QSOX2 TMD. Further, either LD_50_ value is strongly diminished by point mutations known to disrupt the respective helix-helix interface (Fig. 1b,c). The negative controls GpA G83I and QSOX2 S8A diminish self-interaction of the respective TMDs as described previously^19^,^43^ using the ToxR system and do not affect integration of the TMDs into the inner bacterial membrane. These results confirm the ability of BLaTM to determine sequence-specific TMD-TMD interactions.

The results presented in Fig. 1 were obtained with optimized constructs. In genetic detection systems, like ToxR, the efficiency by which a TMD-TMD interaction is translated into a measurable signal depends on the orientation of the interacting face of the TMD helix relative to the signaling domain^18^,^44^. To identify the optimal orientation of TMDs in BLaTM, we inserted up to three amino acids at the TMD N-terminus and simultaneously removed them at the C-terminus; this shifts the interacting surface of the TMD helix relative to the BLa domains in a stepwise fashion. The results reveal optimal ampicillin resistance as well as wild-type/mutant discrimination for the TMD orientations represented by constructs GpA_+1_ and QSOX2_+2_, respectively (Fig. 1b,c and Supplementary Fig. S4b,c). Further, we tested the impact of the length of the flexible linker connecting TMD and BLa domains. An SGS(GGGS)_2_GS linker gives high LD_50_ values and good wild-type/mutant discrimination and was therefore chosen for all subsequent experiments (Fig. 1b,c and Supplementary Fig. S4a,c). To rule out that the different ampicillin resistances result from differences in protein expression, we performed a control experiment which ascertained that neither the mutations in the TMD, their orientations, or the length of the flexible linker affect the amount of expressed protein, as detected by Western blotting (Supplementary Fig. S4d).

We also assessed the impact of the signal peptide. Exemplified by the GpA and QSOX2 TMDs, expression of BLaTM 1.2 proteins tends to yield somewhat lower LD_50_ values and wild-type/mutant discrimination compared to BLaTM 1.1, depending on the TMD orientation (Supplementary Fig. S5). Yet, BLaTM 1.2 proteins were undetectable by Western blotting (data not shown) suggesting that only a fraction of the readily detectable BLaTM 1.1 proteins (Supplementary Fig. S4d) contributes to ampicillin resistance.

**Figure 2:**
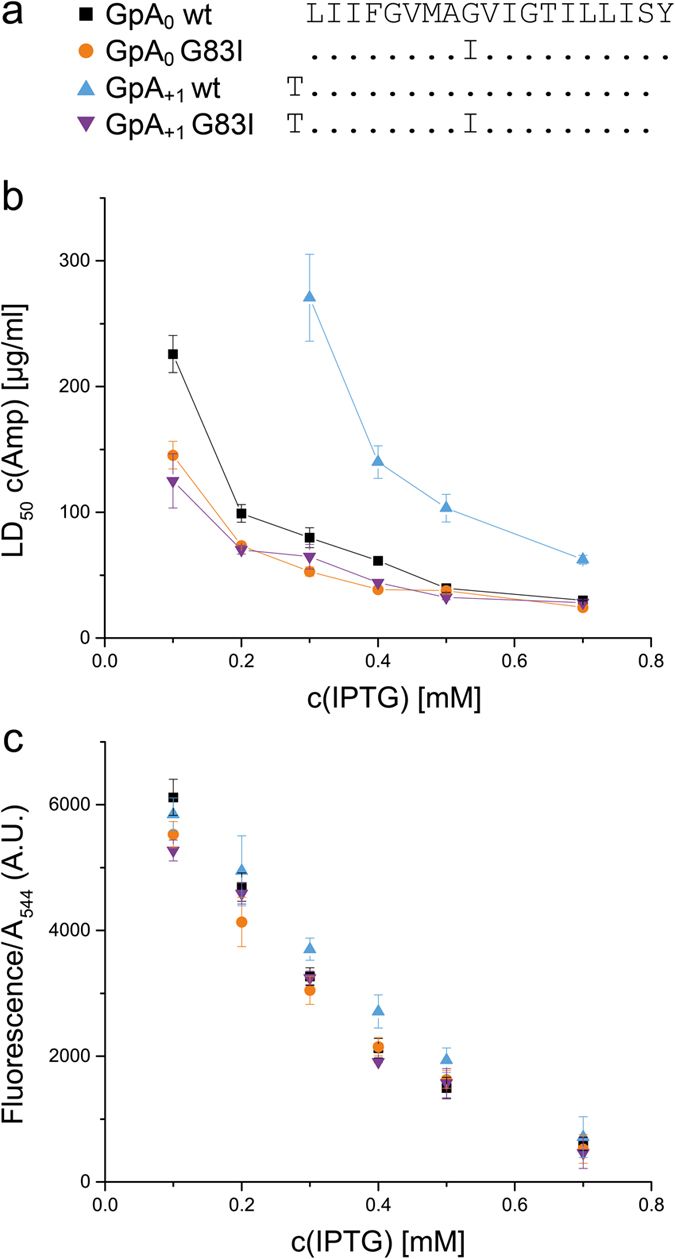
Influence of expression level on ampicillin resistance and wild-type/mutant discrimination. (**a**) GpA wild-type and mutant TMD frames tested. (**b**) Effect of IPTG concentration on ampicillin resistance. Lowering the activity of the pBAD promoter by increasing the IPTG concentration^45^ (133 μM arabinose) influences LD_50_ in a way that depends on TMD orientation. No data points are given for GpA_+1_ wt at 0.1 mM and 0.2 mM IPTG since the corresponding LD_50_ values were too high for reliable determination. (**c**) For control, protein expression was analyzed by GFP fluorescence that was normalized to cell density (A_544_, A.U. = arbitrary units). Even if the GFP moiety is proteolytically cleaved off from part of the BLa proteins during expression (see: Supplementary Fig. S4d), GFP fluorescence represents the amount of originally expressed protein. Note that the expression level varies as a function of induction but not of TMD orientation or sequence. All TMDs are expressed in BLaTM 1.2 in *E. coli* JM83 where the expression level is too low for detection by Western blotting. Means ± SEM, n = 3.

BLaTM 1.2 was then used to assess the influence of the expression level, as detected by the GFP fluorescence of the hybrid proteins, on the LD_50_ of two different TMD orientations (GpA_0_ and GpA_+1_). The pBAD promoter is negatively regulated by Isopropyl-thiogalactopyranoside (IPTG)^45^. Indeed, decreasing IPTG levels led to a progressive 4-fold to 7-fold increase in LD_50_ which is accompanied by the expected increase in expression level. The results also show that the power of BLaTM to discriminate between the LD_50_ values conferred by wild-type and mutant TMDs depends on the expression level, which is in accord with the law of mass action. Specifically, the low wild-type/mutant discrimination seen with construct GpA_0_ at a high IPTG concentration increases substantially by lowering IPTG (Fig. 2).

To validate BLaTM for heterotypic TMD-TMD interactions, we employed the LS46 model TMD previously isolated from a combinatorial library^32^. Using the ToxR assay, it had been shown that LS46 shows strong homotypic interaction. This interaction was largely preserved when the essential interfacial residues were grafted onto a poly-Leu backbone (L19GG_D_6_R_7_) revealing that it mainly rests on ionizable amino acids in combination with a GxxxG motif. Here, we confirm similar homotypic interaction of LS46 and L19GG_D_6_R_7_ with BLaTM (Fig. 1d, green and orange bars). Importantly, deconstructing LS46 into model TMDs holding either Asp or Arg and co-expressing them (L19GG_D_6_/L19GG_R_7_) results in even higher LD_50_ values (brown bars). Conversely, co-expressing TMDs with like charges (blue bars) confers only low ampicillin resistance. We conclude that the strong heterotypic interaction results from electrostatic force between oppositely charged amino acid side chains. We also note that the LD_50_ is independent on whether Arg or Asp are located on the N-BLa TMD or on the C-BLa TMD, respectively (Fig. 1d). For additional controls, strong homodimers (GpA_+1_, LS46) as well as the weak GpA_+1_G83I homodimer were tested for non-specific heterodimer formation with other TMDs, including GpA_+1_G83I, an artificial poly-leucine sequence (L16, LLLLLLLLLLLLLLLL), the human leukocyte antigen β-subunit TMD (HLADQB, SGIGGFVLGLIFLGLGLII), TM5 of the Epstein-Barr virus latent membrane protein 1 (LMP, LLAFFLAFFLDLILLIIALY), and the QSOX2 TMD (SLCVVLYVASSLFLMVMYFF). Figure S6 shows that these heterotypic configurations elicited only around 50% of the LD_50_ values of the homodimers, thus corroborating the specificity of the assay.

To validate that BLaTM can be used to isolate high-affinity TMDs from combinatorial libraries, we established that the assay can discriminate high- and low-affinity TMDs based on the differential survival of bacteria on selective agar plates (schematized in Supplementary Fig. S3b). Specifically, after plating equal numbers of cells expressing GpA_+1_ or the respective G83I mutant on agar, containing 133 μM arabinose for induction plus 5 μg/mL ampicillin for selection, the number of colonies expressing the high-affinity wild-type GpA_+1_ outnumbered the colonies with the low-affinity mutant by a factor of five. A subsequent selection step where the bacteria surviving on plates were subjected to a second round of selection in liquid medium (133 μM arabinose plus 30 μg/mL ampicillin) increased the resolution to a factor of 30.

In sum, BLaTM 1.1 and BLaTM 1.2 are both suited to investigate sequence-specific homo- and heterotypic TMD-TMD interactions in a bacterial membrane. Further, the system enables the isolation of high-affinity TMDs based on survival of expressing cells in selective medium.

### Identification of heterotypic TMD-TMD interactions based on polar interactions

**Figure 3:**
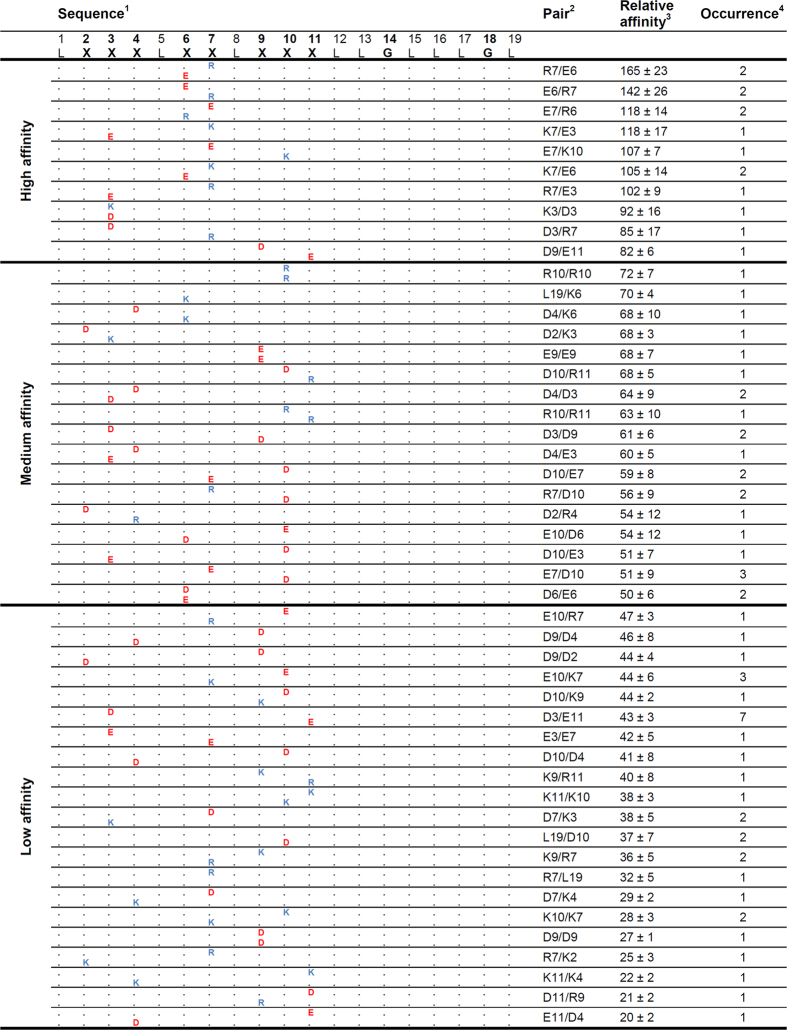
List of isolated TMD pairs sorted by affinity. ^1^The sequence above the identified sequence pairs represents the randomized model based on L19GG (X = R, K, E, or D). Dots in the identified sequences represent Leu. ^2^Single letter code of isolated N-BLa (upper sequence)/C-BLa (lower sequence) combination. ^3^In % (GpA_+1_ wt = 100%; GpA_+1_ G83I = 29 ± 3%; L19GG = 37 ± 4%); means ± SEM, n = 4–12. ^4^Number of times the sequence pair was found in the selected TMDs.

**Figure 4:**
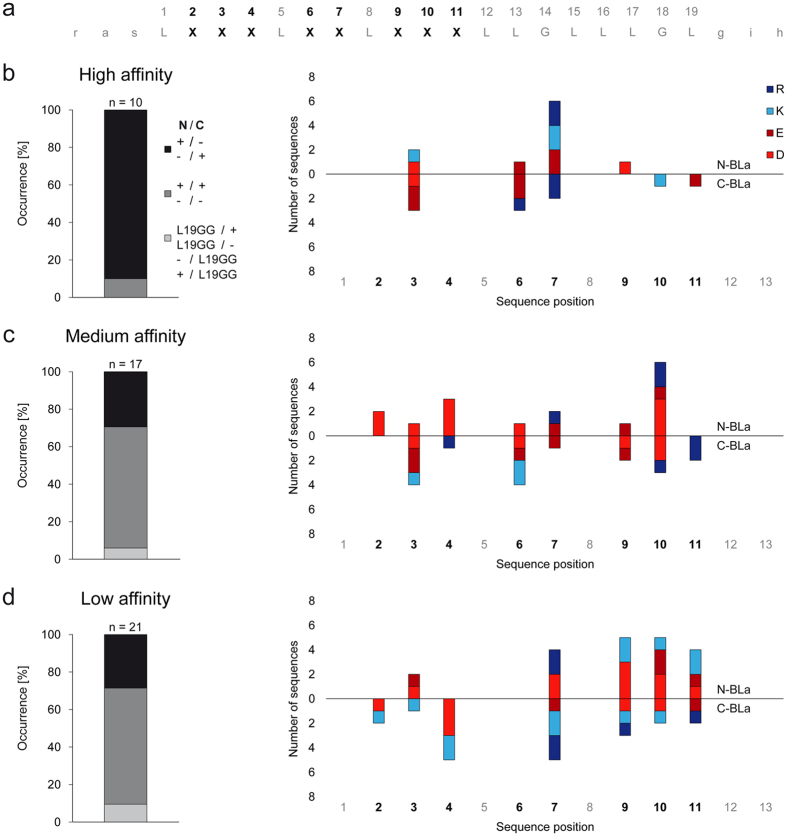
Design of combinatorial library and analysis of TMD pairs. (**a**) Pattern of randomized positions within the L19GG background. Each sequence contains a single Glu, Asp, Lys, or Arg at one of the positions X. Flanking sequences (lower case) represent the *Nhe*I/*Bam*HI restriction sites. (**b–d**) An evaluation of the TMD pairs that were isolated by growth on ampicillin and sorted into high, medium and low affinity based on their individually measured LD_50_ values (Fig. 3). Left panels: Occurrence of pairs with oppositely charged (+/−, −/+; black bars) or like charged (+/+, −/−; grey bars) residues as well as pairs where only one TMD contained an ionizable residue (light grey). Right panels: Distribution of ionizable residues along the isolated TMD sequences encoded by N-BLa (upward pointing bars) or C-BLa (downward pointing bars) plasmids. Note that high-affinity TMD pairs tend to have oppositely charged residues mainly at positions 3, 6, and 7 and that positively and negatively charged amino acids are found in TMDs encoded by N-BLa as well as by C-BLa plasmids.

**Figure 5:**
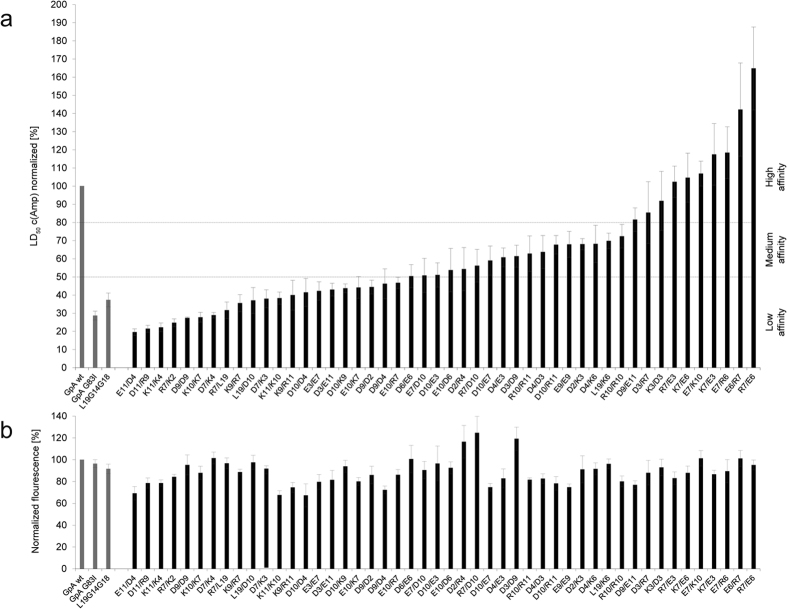
Selection of TMD pairs from a combinatorial library. (**a**) LD_50_ values were determined using BLaTM 1.2 in *E. coli* JM83 cells and are shown relative to wild-type GpA_+1_ (=100%) as listed in Fig. 3. Protein expression was induced with 133 μM arabinose plus 0.3 mM IPTG. Horizontal broken lines separate low-affinity pairs (<50% GpA) from medium-affinity (50% to 80% GpA) and high-affinity (>80% GpA) pairs. (**b**) For control, protein expression levels were analyzed by GFP fluorescence normalized to cell density and wild-type GpA_+1_ (=100%). Note that the expression level does not increase with increasing LD_50_; therefore, higher ampicillin resistance is caused by specific TMD interactions, rather than by increased protein expression. Means ± SEM, n = 4–12.

In a practical application of BLaTM, we sought to identify novel patterns of ionizable residues that can drive heterotypic TMD-TMD interactions. To this end, we designed a focussed combinatorial TMD library based on the poly-Leu template with an invariant GxxxG (L19GG). In each TMD of this library, a single position X of an XXX.XX.XXX pattern corresponds to an Asp, Glu, Lys, or Arg residue (Figs 3 and 4a). This pattern expands the X.XX.XX motif used in our previous screen for homotypic interactions where most positions X had been occupied by multiple ionizable residues in high-affinity TMDs^32^. We thus generated 32 permutations of the L19GG template in N-BLa 1.2 and in C-BLa 1.2. *E. coli* cells were co-transformed with a mixture of the corresponding plasmids. A 10-fold excess of the number of transformants over the number of possible sequences was generated to ensure complete coverage of sequence space. The resulting library was plated under selective pressure and 71 TMD pairs from surviving cells were sequenced, of which 46 were unique. The LD_50_ value of each pair was quantified and normalized to the LD_50_ of the GpA_+1_ homodimer. Isolates were grouped into ‘high-affinity’ (>80% GpA), ‘medium-affinity’ (50% to 80% GpA), and ‘low-affinity’ (<50% GpA) TMD pairs (Figs 3, 4 and 5a). To exclude that the different LD_50_ values originate from different expression levels, we ascertained in a control experiment that the GFP fluorescences are comparable and do not correlate with affinity (Fig. 5b).

An analysis of the results yields the following: First, ~90% of high-affinity TMD pairs contain oppositely charged residues. In medium-affinity and low-affinity TMD pairs, this number drops to <30% while pairs with like charges predominate (Fig. 4b–d, left panels). Second, ionizable residues predominate at positions 3, 6, and 7 in high-affinity pairs while being spread out more evenly in medium- and low-affinity pairs (Fig. 4b–d, right panels). Third, high-affinity pairs with oppositely charged residues at positions 6 and 7 are overrepresented in the isolates; specifically, eight TMD pairs with this pattern were identified (Fig. 3) which exceeds their expected random abundance (1.04 pairs) by a factor of eight. Although the statistical significance of this finding is difficult to ascertain given the low total number of isolates, it is striking that none of the medium- and low affinity TMD pairs contains oppositely charged residues at positions 6 and 7. Fourth, the ionizable residues of a pair are within five residue positions of each other in 100% of the high-affinity and 88% of medium affinity cases, but in only 67% of their low-affinity counterparts (Fig. 3). We note that multiple inverse TMD pairs beyond the E6/R7 and R6/D7 pairs were not identified here; this suggests that a complete coverage of TMD sequence space by more exhaustive selection experiments is likely to produce additional TMD pairs interacting by electrostatic force.

## Discussion

With BLaTM, we have developed a novel genetic tool to investigate homo- and heterotypic TMD-TMD interactions in a natural membrane. We present two versions of the system that differ by the nature of the N-terminal signal peptide. Both versions faithfully report sequence-specific interactions; the lower expression level associated with BLaTM 1.2 may make this version better suited for regulating the density of the TMDs in the bacterial membrane. BLaTM has several advantages over existing methods reporting heterotypic TMD-TMD interactions, such as GALLEX, BACTH, or MaMTH^34^,^36^,^37^,^38^,^40^: (a) TMDs adopt an N_out_ TM topology which corresponds to the natural topology exhibited by the majority of single-span membrane proteins; (b) only membrane-integrated hybrid protein, but not protein retained in the cytoplasm, can contribute to ampicillin resistance; and (c) since no chromosomally integrated reporter genes are required, any *E. coli* strain can be used, allowing measurements in strains with non-natural lipid compositions^46^.

We demonstrate the practical applicability of BLaTM by screening a focussed combinatorial library for TMDs dimerized by ionic interactions. Previously, a database analysis has uncovered hundreds of natural TMDs from single-span membrane proteins that contain either a basic or an acidic residue plus GxxxG^32^. These TMDs might thus enter heterotypic interactions if the respective proteins are co-expressed in a spatio-temporal manner. Comparing the sequences of high-, medium-, and low-affinity TMD pairs, as isolated in the present study, revealed a number of structural requirements for polar interactions within the membrane. Accordingly, ionizable amino acids can contribute to heterotypic TMD-TMD interactions most efficiently if they are of opposite charge and located about two helical turns upstream (i.e., at positions 6 and 7) of the GxxxG motif that was previously shown to support them^32^. High-affinity TMDs containing ionizable residues at this location are overrepresented relative to expectation which confirms the power of BLaTM to select for strongly interacting pairs. Presumably, the interaction between basic and acidic residues is driven by electrostatic forces. By contrast, the heterotypic interaction between TMDs both of which contain either a basic or an acidic residue is attributed to hydrogen bonding between the respective side chains. Interhelical hydrogen bonding between polar residues has previously been implied in homotypic TMD-TMD interactions^47^,^48^. In general, helix-helix interaction is promoted by a close spacing of ionizable residues (i), allowing for (i) to (i′) or (i′ ± 1, 2, 3, 4) bonding. In cases where significant affinity is seen despite a large spacing of ionizable amino acids, hydrogen bonds between side chains and the helix backbone of the partner are likely to promote the interaction. Such side-chain/main-chain hydrogen bonds frequently occur between TMDs of multi-span membrane proteins^49^. Although we focussed on isolating high-affinity TMD pairs in this study, we note an unexpected abundance of low-affinity pairs in the isolates. This is attributed to the prevalence of the low-affinity pairs in the library leading to spill-over in the selection procedure. We present these sequences since, surprisingly, several low-affinity pairs (E10/R7, R10/K7, D10/K9, D7/K3, D7/K4) contain closely spaced residues of opposite charge which is one of the hallmarks of our high-affinity pairs. This indicates that the basic factors describing efficient ionic interactions, as outlined above, are required but not sufficient. Presumably, high TMD-TMD affinity may require a productive interplay between residue spacing, side-chain geometry, helix-helix crossing angle, depth of membrane insertion etc.

## Methods

### Plasmid design and construction

N-BLa and C-BLa hybrid proteins are separately encoded by two low-copy plasmids. The N-BLa plasmid contains a p15A origin^50^ and encodes chloramphenicol acetyltransferase for chloramphenicol (Cm) resistance. The C-BLa plasmid is derived from the pBAD322K plasmid (GenBank: DQ119285.1, kindly provided by Dr. John Cronan, University of Illinois) which contains a pBR322 origin and encodes AraC and aminoglycoside 3′-phosphotransferase for kanamycin (Kan) resistance^51^. The non-coding region from nucleotides 4246–4454/0–2 was deleted by Q5 mutagenesis (NEB) for cloning purposes. Since these plasmids have similar copy numbers, expression of the reading frames under control of the arabinose promoter pBAD is thus expected to yield equivalent amounts of proteins. The TEM-1 β-lactamase is split into the N-fragment (residues 23–194, N-BLa protein) and the C-fragment (residues 196–286, C-BLa protein)^41^. The M182T mutation, which increases the stability of the enzyme^52^, was introduced by QuikChange mutagenesis (New England Biolabs)^53^. To ensure N_out_ transmembrane topology, both β-lactamase fragments are preceded by a cleavable signal peptide. A pelB signal (UniProtKB accession number Q00205, residues 1–20) is used in BLaTM version 1.1 while an ompA signal (UniProtKB accession number P0A910, residues 1–22) is used in BLaTM version 1.2. These fragments are linked via flexible GGS linkers to the TMD of interest (Fig. S4a). The C-termini of the TMDs are linked via a rigid helical linker (A(EAAAK)_5_A)^54^ to superfolder GFP^55^, a FLAG-epitope is appended to the C-terminus of GFP^56^. To insert TMDs of interest into the plasmids, corresponding synthetic oligonucleotides were hybridized to a cassette, phosphorylated, and ligated into the vectors previously digested by *Nhe*I and *Bam*HI. To prevent religation of parental vectors, ligation mixes were incubated with *Apa*I prior to transformation since the plasmids contain the integrin α5 L1002G/A1003P TMD mutant encoding an *Apa*I restriction site. Supplementary Fig. S1 details the plasmid maps and amino acid sequences of encoded hybrid proteins. Plasmids will be deposited at addgene (https://www.addgene.org/).

### Determining ampicillin LD_50_ values

The general work flow for investigating the interaction of candidate TMDs is outlined in Supplementary Fig. S3a. Competent *E. coli* BL21 or JM83 cells were co-transformed with N-BLa and C-BLa plasmids containing a given TMD pair and grown overnight at 37 °C on LB-agar plates containing 34 μg/mL Cm and 35 μg/mL Kan for plasmid inheritance. After incubation for 14 h to 18 h at 37 °C, colonies were either picked for immediate use or the plates were sealed with Parafilm (Pechiney Plastic Packaging) and stored at 4 °C for up to one week. Overnight cultures were conducted by inoculating 5 mL of LB-medium (Cm, Kan) with 10 colonies from one agar plate, followed by incubation in a turning wheel for 14 h to 18 h at 37 °C. An expression culture was started with a 1:10 dilution of the overnight culture in 5 mL expression medium containing arabinose. For BLaTM 1.1, this consisted of LB-medium (Cm, Kan) with 1.33 mM arabinose. For BLaTM 1.2, this consisted of LB-medium (Cm, Kan) with 133 μM arabinose and 0.1 mM–0.7 mM IPTG. After exactly 4 h at 37 °C, the expression cultures were diluted to an OD_600_ = 0.1 in expression medium. To expose the bacteria to different ampicillin concentrations, an LD_50_ culture was prepared by pipetting 2 mL of the diluted expression culture into each cavity of a 12-well plate (Greiner Bio-one). Freshly prepared ampicillin stock (5 to 40 mg/mL in water) was added, resulting in ampicillin concentrations ranging from 0 to 600 μg/mL, depending on the affinity of the TMD under investigation. As a rule, the maximum ampicillin concentration to be used for a particular case should be about twice the mean LD_50_. The plates were incubated in a moisturized container for 19 h at 37 °C and 200 rpm on a shaker (Shaking amplitude 10 mm, Orbital Shaker 3005, GFL). Cell density was measured via absorbance at 544 nm in a microplate reader (FluoStar, BMG Labtech). To minimize clonal variation, at least two transformations were done and at least two separate LD_50_ cultures were inoculated from each batch of transformed bacteria using ten colonies for each culture. Thus, at least 40 colonies entered each determination of LD_50_. To measure and collect LD_50_ values from the dose-response curves, we developed ECCpy, an open-source program in python (https://github.com/teese/eccpy). ECCpy fits the data points with the Hill equation (eq. (1))^57^ where c is the maximum of the sigmoidal curve, x is the ampicillin concentration, k is the LD_50_ and g is the Hill coefficient).


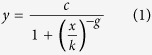


### Western blotting and GFP fluorescence measurement

With BLaTM 1.1, expression levels of the BLaTM proteins were determined by Western blot analysis, with an antiserum recognizing the FLAG-epitope (ANTI-FLAG M1 antibody, F3040, Sigma-Aldrich). With BLaTM 1.2 constructs, protein expression levels were too low for detection by Western blotting, even with sensitive chemiluminescence methods (data not shown). With BLaTM 1.2 therefore, GFP fluorescence (λ_Ex_ = 485 nm, λ_Em_ = 520 nm, FluoStar, BMG Labtech) was directly measured in the cultures after 4 h expression in 12-well plates. Background fluorescence of pure LB medium was subtracted, and values were normalized to the cell density in the sample by dividing by the absorbance at 544 nm.

### Nitrocefin assay

To measure BLa activity by nitrocefin turnover, 5 mL LB medium (Kan and/or Cm, 1.33 mM arabinose) were inoculated with a 1:10 dilution of an overnight culture of plasmid-containing *E. coli* JM83 cells. After 4 h expression, the cells were washed with sodium phosphate buffer (100 mM, pH 7.0), and 20 μL were used for the enzyme activity assay as previously described^41^.

### Selection of heterotypically interacting TMDs from a combinatorial library

Using the cassette-cloning method described above, a series of BLaTM 1.2 TMD variants were prepared, each containing a single charged residue (Asp, Glu, Arg, or Lys) in the L19GG TMD background, at positions 2, 3, 4, 6, 7, 9, 10, or 11 (Fig. 4a). All constructs were verified by sequencing. Equal amounts of all N-BLa and C-BLa plasmids were combined and electro-competent *E. coli* JM83 cells^58^ were transformed with 1 μg of the plasmid mix. Transformed cells were plated on LB (Cm, Kan) agar containing 133 μM arabinose to induce protein expression. Plates were incubated at 37 °C for 19 h. Each N-BLa and C-BLa plasmid had 33 variants, due to 32 TMDs with charged residues plus the original L19GG, leading to 1089 possible TMD pairs in the full library. To ensure representation of all TMD pairs in the resulting library, we generated a 10-fold excess of transformants on a standard agar plate of diameter 90 mm. Co-transformants were scraped from the agar and resuspended in 5 mL of LB medium (Cm, Kan) containing 133 μM arabinose. Aggregated cells were separated by vigorous vortexing for 1 min. Cultures were then adjusted to a cell density of A_600_ = 0.25 by dilution with medium.

A two-step selection for resistance to ampicillin was carried out, the first on solid media, and the second in liquid media. For the first step, 25 μL of cells were plated on LB-agar (Cm, Kan) containing 133 μM arabinose, and 5 μg/mL ampicillin. Plates were incubated at 37 °C in a sealed container for up to 3 days and surviving colonies were picked. All plates were freshly prepared and dried for 1 h in a laminar flow hood at room temperature. Special care was taken that the surface of the plates was completely dry and the cells were spread equally. We note that higher ampicillin concentrations might be used to obtain a higher fraction of high-affinity TMD pairs in the isolates at the expense of the diversity of obtained sequences. In a second step, each surviving colony was then used to inoculate 4 mL of liquid medium consisting of LB (Cm, Kan) containing 133 μM arabinose, and 30 μg/mL ampicillin, which was grown for 18 h at 37 °C. Plasmids were isolated from cultures that grew to high density, and the respective TMD-coding regions were sequenced using the primers N-BLa_fw (CACGACGCCTGTAGCAATG) and C-BLa_fw (CGAAATAGACAGATCGCTG). In parallel to selecting colonies containing unknown TMDs from combinatorial libraries, we routinely plated bacteria expressing the high-affinity wild-type GpA TMD or its low-affinity G83I mutant on separate plates. If the number of surviving bacteria encoding the wild-type GpA TMD outnumbers the number of cells encoding the mutant by a factor of about five, this control experiment indicates that the experimental conditions are conducive to successful selection.

### Calculation of overrepresentation

Due to randomization with four ionizable amino acids, the probability to isolate a TMD pair with oppositely charged residues in either TMD equals 4*4/1089 = 0.0147. The expected number of such TMD pairs present within the 71 pairs isolated in our screen thus equals 0.147*71 = 1.04. Since we isolated eight pairs where positions 6 or 7 are occupied by oppositely charged residues, these combinations are overrepresented by a factor of eight.

## Additional Information

**How to cite this article**: Schanzenbach, C. *et al*. Identifying ionic interactions within a membrane using BLaTM, a genetic tool to measure homo- and heterotypic transmembrane helix-helix interactions. *Sci. Rep.* **7**, 43476; doi: 10.1038/srep43476 (2017).

**Publisher's note:** Springer Nature remains neutral with regard to jurisdictional claims in published maps and institutional affiliations.

## Supplementary Material

Supplementary Material

## References

[b1] Neumann J., Klein N., Otzen D. E. & Schneider D. Folding energetics and oligomerization of polytopic alpha-helical transmembrane proteins. Archives of Biochemistry and Biophysics 564, 281–296 (2014). 25057769 10.1016/j.abb.2014.07.017

[b2] Li E., Wimley W. C. & Hristova K. Transmembrane helix dimerization: Beyond the search for sequence motifs. Biochimica Et Biophysica Acta-Biomembranes 1818, 183–193 (2012). 10.1016/j.bbamem.2011.08.031 PMC3253898 21910966

[b3] Cymer F., Veerappan A. & Schneider D. Transmembrane helix-helix interactions are modulated by the sequence context and by lipid bilayer properties. Biochim Biophys Acta 1818, 963–973 (2012). 21827736 10.1016/j.bbamem.2011.07.035

[b4] Teese M. G. & Langosch D. Role of GxxxG Motifs in Transmembrane Domain Interactions. Biochemistry 54, 5125–5135 (2015). 26244771 10.1021/acs.biochem.5b00495

[b5] Fink A., Sal-Man N., Gerber D. & Shai Y. Transmembrane domains interactions within the membrane milieu: Principles, advances and challenges. Biochimica et Biophysica Acta 1818, 974–983 (2012). 22155642 10.1016/j.bbamem.2011.11.029

[b6] Walther T. H. & Ulrich A. S. Transmembrane helix assembly and the role of salt bridges. Curr Opin Struct Biol 27, 63–68 (2014). 24907460 10.1016/j.sbi.2014.05.003

[b7] Bano-Polo M. . Polar/Ionizable residues in transmembrane segments: effects on helix-helix packing. PLoS One 7, e44263 (2012). 22984481 10.1371/journal.pone.0044263 PMC3440369

[b8] Illergard K., Kauko A. & Elofsson A. Why are polar residues within the membrane core evolutionary conserved? Proteins 79, 79–91 (2011). 20938980 10.1002/prot.22859

[b9] Call M. E. & Wucherpfennig K. W. Common themes in the assembly and architecture of activating immune receptors. Nature reviews. Immunology 7, 841–850 (2007). 10.1038/nri2186 17960150

[b10] Feng J., Call M. E. & Wucherpfennig K. W. The assembly of diverse immune receptors is focused on a polar membrane-embedded interaction site. PLoS Biol 4, e142 (2006). 16623599 10.1371/journal.pbio.0040142 PMC1440944

[b11] Knoblich K. . Transmembrane Complexes of DAP12 Crystallized in Lipid Membranes Provide Insights into Control of Oligomerization in Immunoreceptor Assembly. Cell Reports 11, 1184–1192 (2015). 25981043 10.1016/j.celrep.2015.04.045 PMC4449314

[b12] Zhang L. . Contribution of hydrophobic and electrostatic interactions to the membrane integration of the Shaker K^+^ channel voltage sensor domain. Proc Natl Acad Sci USA 104, 8263–8268 (2007). 17488813 10.1073/pnas.0611007104 PMC1899110

[b13] DeCaen P. G., Yarov-Yarovoy V., Zhao Y., Scheuer T. & Catterall W. A. Disulfide locking a sodium channel voltage sensor reveals ion pair formation during activation. Proc Natl Acad Sci USA 105, 15142–15147 (2008). 18809926 10.1073/pnas.0806486105 PMC2567506

[b14] MacKenzie K. R. & Fleming K. G. Association energetics of membrane spanning alpha-helices. Curr. Opin. Chem. Biol. 18, 1–8 (2008). 10.1016/j.sbi.2008.04.007 PMC7127525 18539023

[b15] Tome L., Steindorf D. & Schneider D. Genetic systems for monitoring interactions of transmembrane domains in bacterial membranes. Methods in molecular biology (Clifton, N.J.) 1063, 57–91 (2013). 10.1007/978-1-62703-583-5_4 23975772

[b16] Hong H., Blois T. M., Cao Z. & Bowie J. U. Method to measure strong protein-protein interactions in lipid bilayers using a steric trap. Proceedings of the National Academy of Sciences of the United States of America 107, 19802–19807 (2010). 21041662 10.1073/pnas.1010348107 PMC2993359

[b17] Khadria A. S. & Senes A. Fluorophores, Environments, and Quantification Techniques in the Analysis of Transmembrane Helix Interaction Using FRET. Biopolymers 104, 247–264 (2015). 25968159 10.1002/bip.22667 PMC4516689

[b18] Langosch D. L., Brosig B., Kolmar H. & Fritz H.-J. Dimerisation of the glycophorin A transmembrane segment in membranes probed with the ToxR transcription activator. J. Mol. Biol. 263, 525–530 (1996). 8918935 10.1006/jmbi.1996.0595

[b19] Russ W. P. & Engelman D. M. TOXCAT: A measure of transmembrane helix association in a biological membrane. Proc. Natl. Acad. Sci. USA 96, 863–868 (1999). 9927659 10.1073/pnas.96.3.863 PMC15316

[b20] Elazar A. . Mutational scanning reveals the determinants of protein insertion and association energetics in the plasma membrane. Elife 5 (2016). 10.7554/eLife.12125 PMC4786438 26824389

[b21] Armstrong C. R. & Senes A. Screening for transmembrane association in divisome proteins using TOXGREEN, a high-throughput variant of the TOXCAT assay. Biochimica Et Biophysica Acta-Biomembranes 1858, 2573–2583 (2016). 10.1016/j.bbamem.2016.07.008 PMC5045792 27453198

[b22] Russ W. P. & Engelman D. M. The GxxxG motif: a framework for transmembrane helix-helix association. J. Mol. Biol. 296, 911–919 (2000). 10677291 10.1006/jmbi.1999.3489

[b23] Sal-Man N., Gerber D. & Shai Y. The identification of a minimal dimerization motif QXXS that enables homo- and hetero-association of transmembrane helices *in vivo*. J. Biol. Chem. 280, 27449–27457 (2005). 15911619 10.1074/jbc.M503095200

[b24] Dawson J. P., Weinger J. S. & Engelman D. M. Motifs of serine and threonine can drive association of transmembrane helices. J. Mol. Biol. 316, 799–805 (2002). 11866532 10.1006/jmbi.2001.5353

[b25] Sal-Man N., Gerber D., Bloch I. & Shai Y. Specificity in Transmembrane Helix-Helix Interactions Mediated by Aromatic Residues. J. Biol. Chem. 282, 19753–19761 (2007). 17488729 10.1074/jbc.M610368200

[b26] Ridder A., Skupjen P., Unterreitmeier S. & Langosch D. Tryptophan Supports Interaction of Transmembrane Helices. J. Mol. Biol. 354, 894–902 (2005). 16280130 10.1016/j.jmb.2005.09.084

[b27] Johnson R. M., Hecht K. & Deber C. M. Aromatic and cation-pi interactions enhance helix-helix association in a membrane environment. Biochemistry 46, 9208–9214 (2007). 17658897 10.1021/bi7008773

[b28] Zhou F. X., Cocco M. J., Russ W. P., Brunger A. T. & Engelman D. M. Interhelical hydrogen bonding drives strong interactions in membrane proteins. Nature Struct. Biol. 7, 154–160 (2000). 10655619 10.1038/72430

[b29] Choma C., Gratkowski H., Lear J. D. & DeGrado W. F. Asparagine-mediated self-association of a model transmembrane helix. Nature Struct. Biol. 7, 161–166 (2000). 10655620 10.1038/72440

[b30] Unterreitmeier S. . Phenylalanine Promotes Interaction of Transmembrane Domains via GxxxG Motifs. J. Mol. Biol. 374, 705–718 (2007). 17949750 10.1016/j.jmb.2007.09.056

[b31] Herrmann J. . Complex patterns of histidine, hydroxylated amino acids and the GxxxG motif mediate high-affinity transmembrane domain interactions. J. Mol. Biol. 385, 912–923 (2009). 19007788 10.1016/j.jmb.2008.10.058

[b32] Herrmann J. R. . Ionic interactions promote transmembrane helix-helix association depending on sequence context. J Mol Biol 396, 452–461 (2010). 19961858 10.1016/j.jmb.2009.11.054

[b33] Berger B. W. . Consensus motif for integrin transmembrane helix association. Proc Natl Acad Sci USA 107, 703–708 (2010). 20080739 10.1073/pnas.0910873107 PMC2818961

[b34] Su P.-C. & Berger B. W. A novel assay for assessing juxtamembrane and transmembrane domain interactions important for receptor heterodimerization. Journal of molecular biology 425, 4652–4658 (2013). 23876708 10.1016/j.jmb.2013.07.022

[b35] Joce C., Wiener A. A. & Yin H. Multi-Tox: Application of the ToxR-transcriptional reporter assay to the study of multi-pass protein transmembrane domain oligomerization. Biochimica et Biophysica Acta 1808, 2948–2953 (2011). 21791200 10.1016/j.bbamem.2011.07.008 PMC3195870

[b36] Schneider D. & Engelman D. M. GALLEX: a measurement of heterologous association of transmembrane helices in a biological membrane. J.Biol.Chem. 278, 3105–3111 (2003). 12446730 10.1074/jbc.M206287200

[b37] Petschnigg J. . The mammalian-membrane two-hybrid assay (MaMTH) for probing membrane-protein interactions in human cells. Nature methods 11, 585–592 (2014). 24658140 10.1038/nmeth.2895

[b38] Karimova G., Dautin N. & Ladant D. Interaction network among Escherichia coli membrane proteins involved in cell division as revealed by bacterial two-hybrid analysis. J Bacteriol 187, 2233–2243 (2005). 15774864 10.1128/JB.187.7.2233-2243.2005 PMC1065216

[b39] Sawma P. . Evidence for new homotypic and heterotypic interactions between transmembrane helices of proteins involved in receptor tyrosine kinase and neuropilin signaling. J Mol Biol 426, 4099–4111 (2014). 25315821 10.1016/j.jmb.2014.10.007

[b40] Steindorf D. & Schneider D. *In vivo* selection of heterotypically interacting transmembrane helices: Complementary helix surfaces, rather than conserved interaction motifs, drive formation of transmembrane hetero-dimers. Biochim Biophys Acta (2016). 10.1016/j.bbamem.2016.11.017 27915045

[b41] Galarneau A., Primeau M., Trudeau L. E. & Michnick S. W. Beta-lactamase protein fragment complementation assays as *in vivo* and *in vitro* sensors of protein protein interactions. Nat Biotechnol 20, 619–622 (2002). 12042868 10.1038/nbt0602-619

[b42] Alber T. Structure of the leucine zipper. Corr.Op.Genet.and Developm. 2, 205–210 (1992). 10.1016/s0959-437x(05)80275-8 1638114

[b43] Ried C. L., Scharnagl C. & Langosch D. Entrapment of Water at the Transmembrane Helix-Helix Interface of Quiescin Sulfhydryl Oxidase 2. Biochemistry 55, 1287–1290 (2016). 26894260 10.1021/acs.biochem.5b01239

[b44] Ruan W., Becker V., Klingmüller U. & Langosch D. The interface between the self-assembling erythropoietin receptor transmembrane segments corresponds to a heptad repeat pattern. J. Biol. Chem. 279, 3273–3279 (2004). 14602718 10.1074/jbc.M309311200

[b45] Lee S. K. . Directed evolution of AraC for improved compatibility of arabinose- and lactose-inducible promoters. Applied and environmental microbiology 73, 5711–5715 (2007). 17644634 10.1128/AEM.00791-07 PMC2074931

[b46] Shibuya I. Metabolic regulations and biological functions of phospholipids in Escherichia coli. Prog Lipid Res 31, 245–299 (1992). 1287667 10.1016/0163-7827(92)90010-g

[b47] Zhou F. X., Merianos H. J., Brunger A. T. & Engelman D. M. Polar residues drive association of polyleucine transmembrane helices. Proc Natl Acad Sci USA 98, 2250–2255 (2001). 11226225 10.1073/pnas.041593698 PMC30124

[b48] Gratkowski H., Lear J. D. & DeGrado W. F. Polar side chains drive the association of model transmembrane peptides. Proc. Natl. Acad. Sci. USA 98, 880–885 (2001). 11158564 10.1073/pnas.98.3.880 PMC14678

[b49] Zhang S. Q. . The membrane- and soluble-protein helix-helix interactome: similar geometry via different interactions. Structure 23, 527–541 (2015). 25703378 10.1016/j.str.2015.01.009 PMC4351763

[b50] Cozzarelli N. R., Kelly R. B. & Kornberg A. A minute circular DNA from Escherichia coli 15. Proc Natl Acad Sci USA 60, 992–999 (1968). 4875809 10.1073/pnas.60.3.992 PMC225151

[b51] Cronan J. E. A family of arabinose-inducible Escherichia coli expression vectors having pBR322 copy control. Plasmid 55, 152–157 (2006). 16139359 10.1016/j.plasmid.2005.07.001

[b52] Sideraki V., Huang W., Palzkill T. & Gilbert H. F. A secondary drug resistance mutation of TEM-1 beta-lactamase that suppresses misfolding and aggregation. Proc Natl Acad Sci USA 98, 283–288 (2001). 11114163 10.1073/pnas.011454198 PMC14582

[b53] Wang W. & Malcolm B. A. Two-stage PCR protocol allowing introduction of multiple mutations, deletions and insertions using QuikChange Site-Directed Mutagenesis. BioTechniques 26, 680–682 (1999). 10343905 10.2144/99264st03

[b54] Arai R., Ueda H., Kitayama A., Kamiya N. & Nagamune T. Design of the linkers which effectively separate domains of a bifunctional fusion protein. Protein engineering 14, 529–532 (2001). 11579220 10.1093/protein/14.8.529

[b55] Pedelacq J. D., Cabantous S., Tran T., Terwilliger T. C. & Waldo G. S. Engineering and characterization of a superfolder green fluorescent protein. Nat Biotechnol 24, 79–88 (2006). 16369541 10.1038/nbt1172

[b56] Hopp T. P. . A Short Polypeptide Marker Sequence Useful for Recombinant Protein Identification and Purification. Bio-Technol 6, 1204–1210 (1988).

[b57] Hill A. V. The possible effects of the aggregation of the molecules of haemoglobin on its dissociation curves. J Physiol 40, iv–vii (1910).

[b58] Warren D. J. Preparation of highly efficient electrocompetent Escherichia coli using glycerol/mannitol density step centrifugation. Anal Biochem 413, 206–207 (2011). 21362398 10.1016/j.ab.2011.02.036

