# Author Correction: Identifying ionic interactions within a membrane using BLaTM, a genetic tool to measure homo- and heterotypic transmembrane helix-helix interactions

**DOI:** 10.1038/s41598-020-63947-z

**Published:** 2020-04-24

**Authors:** Christoph Schanzenbach, Fabian C. Schmidt, Patrick Breckner, Mark G. Teese, Dieter Langosch

**Affiliations:** Munich Center For Integrated Protein Science (CIPSM) at the Lehrstuhl für Chemie der Biopolymere, Technische Universität München, Weihenstephaner Berg 3, 85354 Freising, Germany

Correction to: *Scientific Reports* 10.1038/srep43476, published online 07 March 2017

This Article and its accompanying supplementary material contain typographical errors in the concentrations of arabinose, where they should be multiplied by a factor of ten. As a result, all instances of ‘133 µM’ should read ‘1.33 mM’ and all instances of ‘1.33 mM’ should read ‘13.3 mM’.

