# Supplementary material for: Identifying ionic interactions within a membrane using BLaTM, a genetic tool to measure homo- and heterotypic transmembrane helix-helix interactions
Source: Sci Rep. 2017 Mar 7;7:43476. doi: 10.1038/srep43476 (PMC5339904; doi:10.1038/srep43476)
Supplement: Supplementary Material [file srep43476-s1.pdf]

Identifying ionic interactions within a membrane using BLaTM, a genetic tool to measure homo- and heterotypic transmembrane helix-helix interactions

Christoph Schanzenbach [1,*], Fabian C Schmidt [1,*], Patrick Breckner [1], Mark G Teese [1], and Dieter Langosch [1]

[1] Munich Center For Integrated Protein Science (CIPS[M]) at the Lehrstuhl für Chemie der Biopolymere, Technische Universität München, Weihenstephaner Berg 3, 85354 Freising, Germany

* These authors contributed equally to this work. Correspondence should be addressed to D.L. (email: langosch@tum.de)

Supplementary Material

Supplementary Figure S1

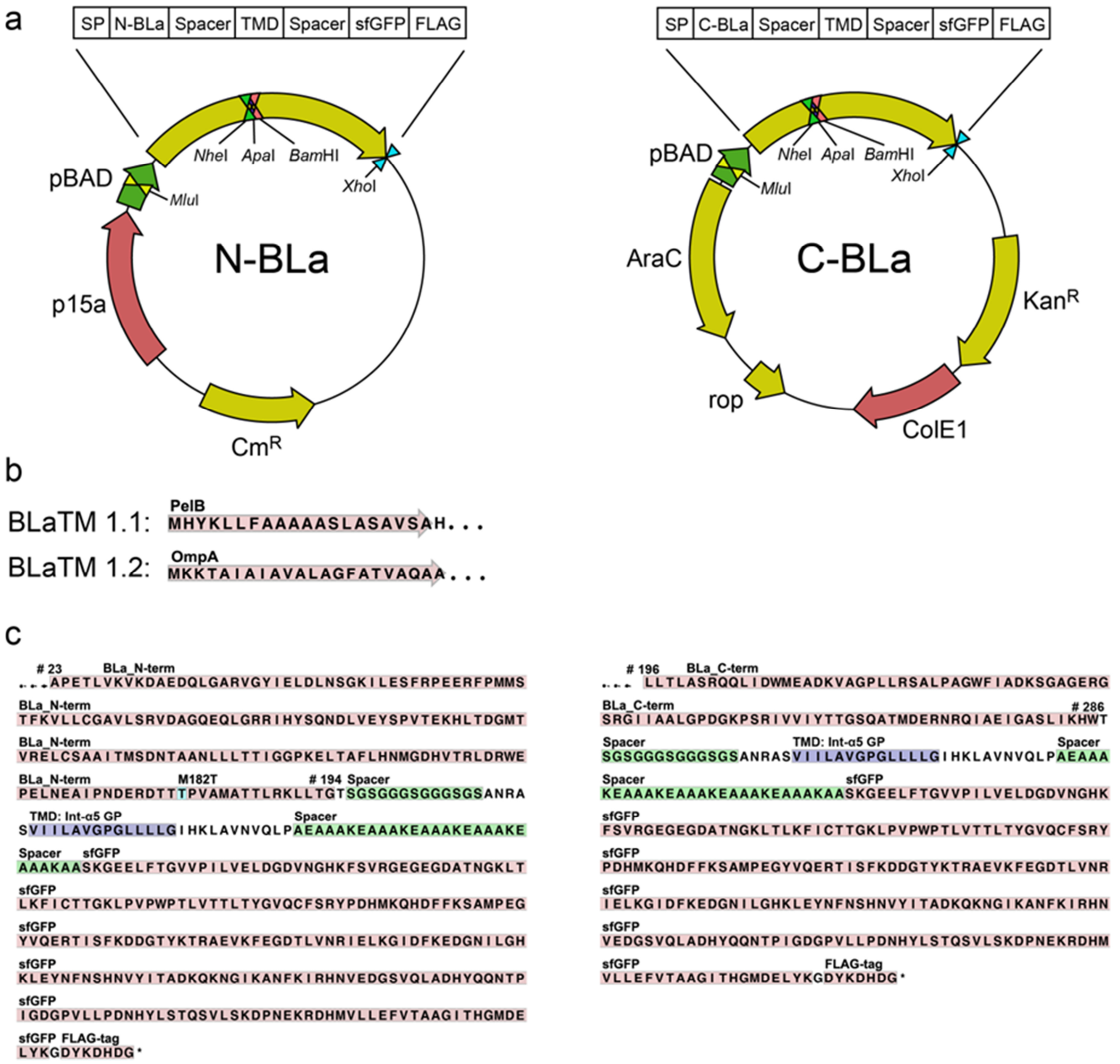

Design of plasmids. (**a**) BLaTM plasmid maps. The unique restriction sites shown are useful for inserting TMD sequences (*Nhe*I, *Bam*HI) or transferring the complete reading frames (*Mlu*I, *Xho*I). (**b**) Signal peptide sequences used in BLaTM 1.1 (pelB) or BLaTM 1.2 (ompA). (**c**) Amino acid sequences of the encoded hybrid proteins with a 13 residue spacer and a TMD sequence containing an *Apa*I restriction site on the genetic level (integrin α5 GP). Numbers at the start of the sequences denote residues of TEM-1 β-lactamase (UniProtKB: P62593).

Supplementary Figure 2

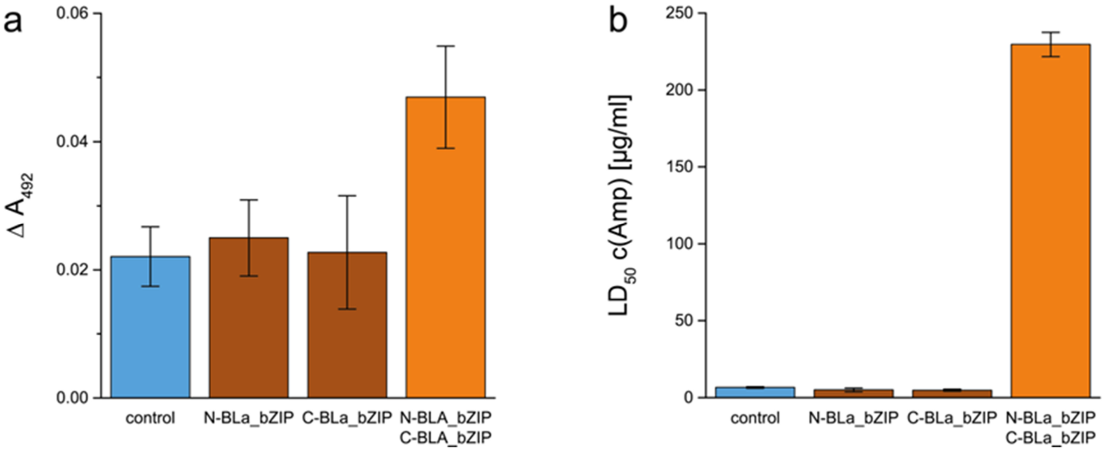

Functional reconstitution of β-lactamase by fusing the strongly self-interacting soluble protein GCN4 to N-BLa and C-BLa fragments. N-BLa or C-BLa proteins were appended to the C-terminus of the GCN4 leucine zipper sequence (bZip) (UniProtKB accession number P03069, residues 235-281) [1] and either expressed alone, or co-expressed in *E. coli* JM83 cells. Enzyme activity in the cells was determined by quantifying the turnover of nitrocefin through the change of A_{492} (**a**) or by determining the ampicillin resistance conferred to the cells (LD_{50}) (**b**). Note that the LD_{50} values discriminate much better between reconstituted enzyme and individually expressed enzyme fragments than the nitrocefin assay.

Supplementary Figure 3

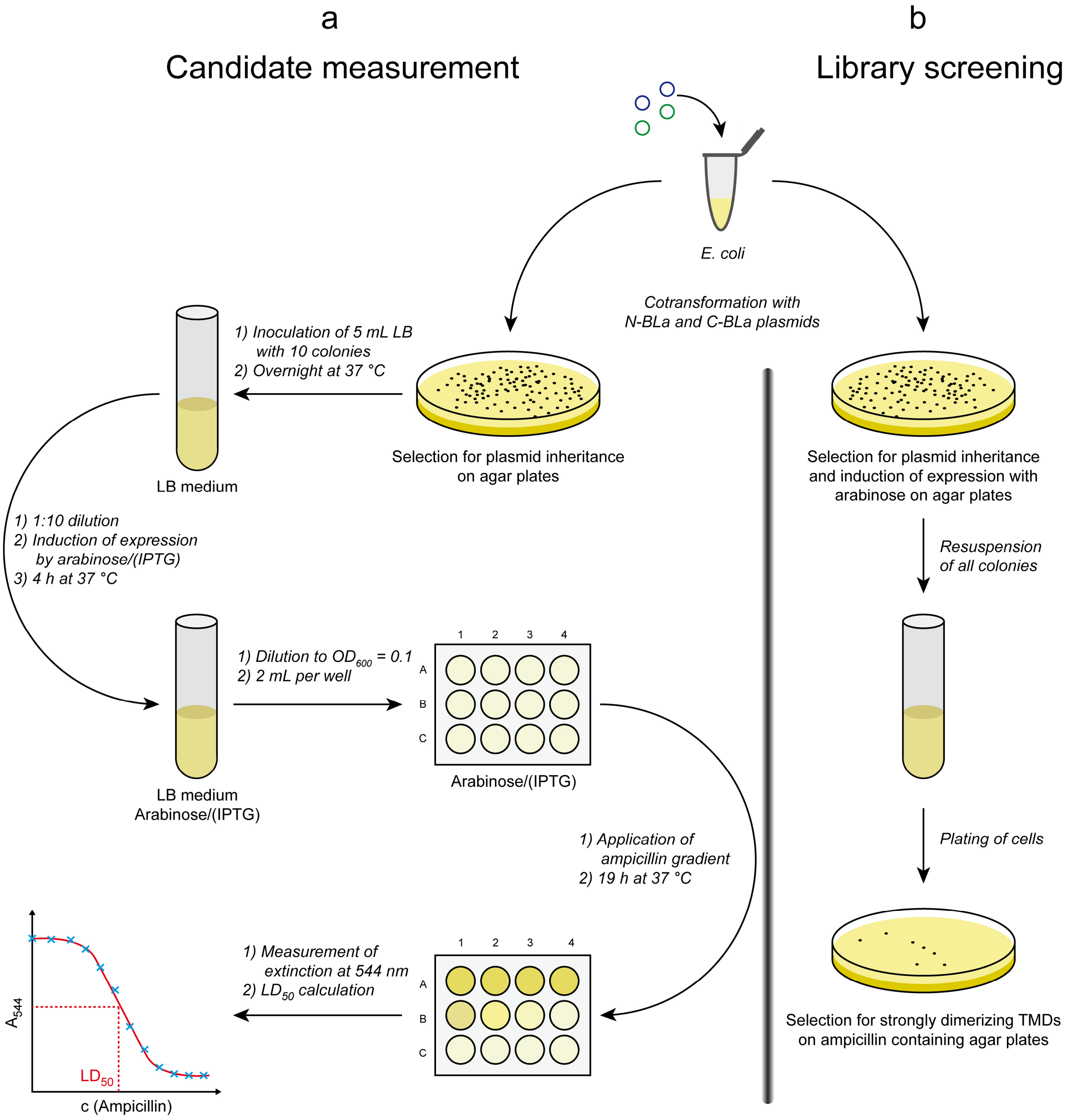

BLaTM workflow used to investigate the interaction of candidate TMDs (**a**) or to select high-affinity TMDs from a combinatorial library (**b**). See Methods for details.

Supplementary Figure 4

a

Linker length	Amino acid sequence
5 aa	SGS GS
9 aa	SGS GGGS GS
13 aa	SGS GGGS GGGS GS
25 aa	SGS GGGS GGGS GGGS GGGS GS

b

Construct	Sequence	Construct	Sequence
GpA$_{-1}$ wt	IIFGVMAGVIGTILLISYA	QSOX2$_{0}$ wt	CVVLYVASSLFLMVMYFFFA
GpA$_{-1}$ G83I	.......I...........	QSOX2$_{0}$ S8A	.......A............
GpA$_{0}$ wt	L...................	QSOX2$_{1}$ wt	L....................
GpA$_{0}$ G83I	L.......I...........	QSOX2$_{1}$ S8A	L.......A............
GpA$_{+1}$ wt	TL...................	QSOX2$_{2}$ wt	SL....................
GpA$_{+1}$ G83I	TL.......I...........	QSOX2$_{2}$ S8A	SL.......A............
GpA$_{+2}$ wt	ITL...................	QSOX2$_{3}$ wt	MSL....................
GpA$_{+2}$ G83I	ITL.......I...........	QSOX2$_{3}$ S8A	MSL.......A............

c

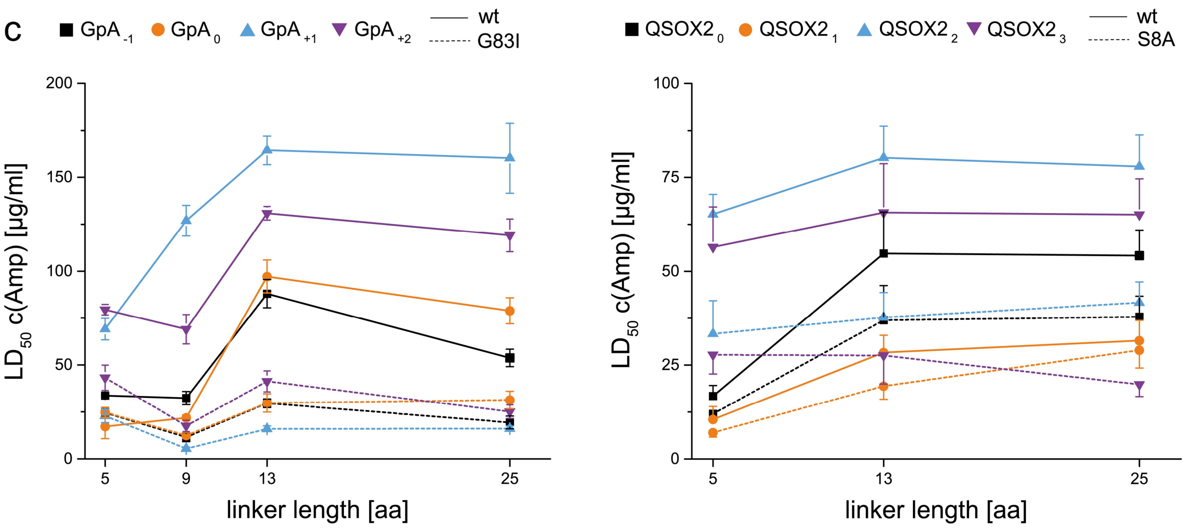

d

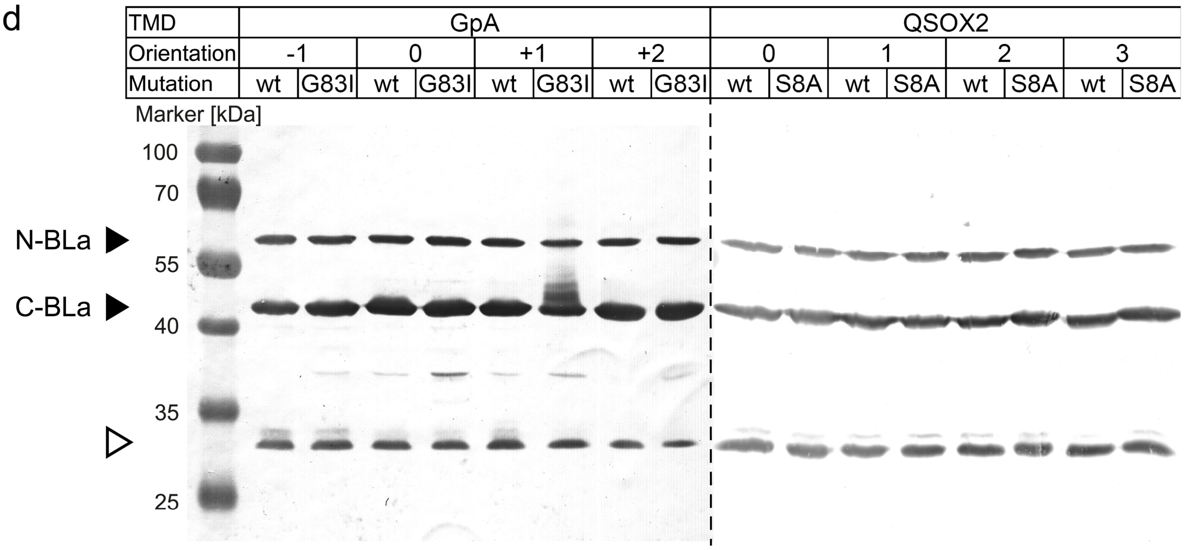

Influence of linker length and TMD orientation on homotypic interaction. (**a**) Sequences of flexible linkers inserted between BLa fragments and the TMD. (**b**) Sequences of GpA and QSOX2 wild-type and mutant TMDs tested in the BLaTM assay. The different sequence frames vary the orientation of the TMD interface relative to the reconstituted β–lactamase. Assuming α–helicity of the TMDs, stepwise insertion of three residues at their N-terminus concurrent with the stepwise deletion of three residues at their C-terminus rotates a TMD-TMD interface by up to 3 × 100°, *i.e.* more than a full helix turn is covered. (**c**) Ampicillin LD_{50} values determined for different TMD orientations as a function of linker length in *E. coli* BL21 cells. Note that a linker length of 13 residues confers good wild-type/mutant discrimination. (**d**) Western blots revealing similar expression of N-BLa and C-BLa proteins (arrows) containing the TMDs inserted at different orientations. The band at ~30 kDa likely corresponds to the cytoplasmic part of the protein (linker plus sfGFP and FLAG-epitope account for 30.2 kDa) presumably produced by proteolysis in the cell. All TMDs were expressed using BLaTM 1.1 in *E .coli* BL21 cells, means ± SEM, n=3-4.

Supplementary Figure 5

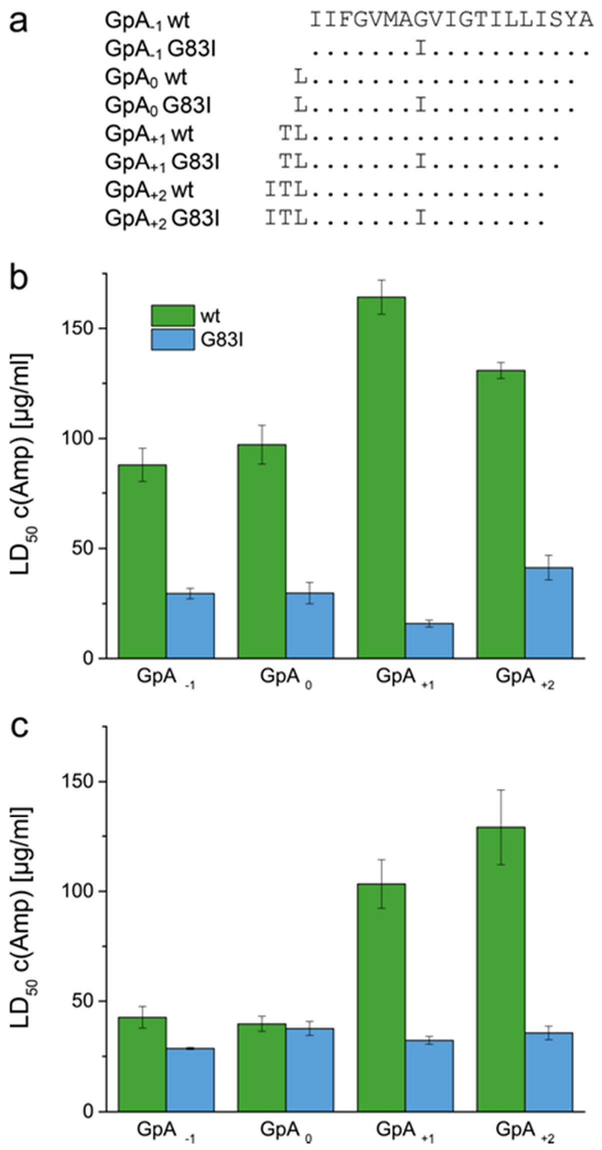

Comparing the impact of the N-terminal signal peptide. (**a**) The different frames of wild-type and mutant GpA TMDs compared here. (**b**) LD_{50} values observed using the pelB signal peptide (BLaTM 1.1, induction by 1.33 mM arabinose, in *E. coli* BL21 cells). (**c**) LD_{50} values observed using the ompA signal peptide (BLaTM 1.2, induction by 133 µM arabinose plus 0.5 mM IPTG, in *E. coli* JM83 cells). Note that LD_{50} values and wild-type/mutant ratios of orientations GpA_{+1} and GpA_{+2} are similar in BLaTM 1.1 (b) and BLaTM 1.2 (c). Lower values and wild-type/mutant ratios are seen with orientations GpA_{-1} and GpA_0 using BLaTM 1.2. This is likely due to a somewhat lower protein concentration in the membrane in (c) since stronger GpA_0 expression (0.1 mM IPTG) increased the wild-type/mutant ratio (Fig. 2). Means ± SEM, n=3-9.

Supplementary Figure 6

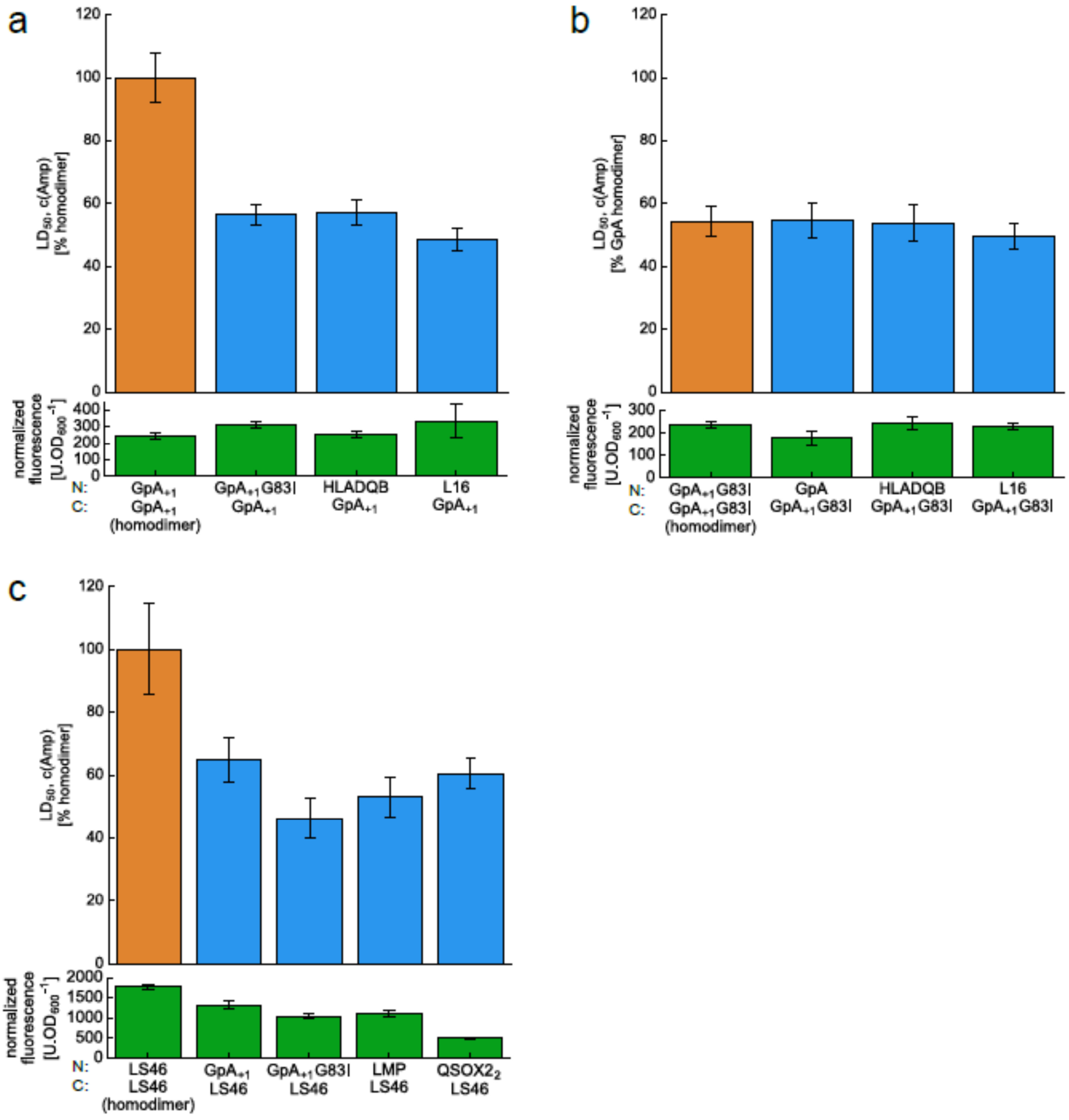

Heterodimerization dimerization signal elicited by non-cognate TMD pairs compared to strong homodimerization signals. **(a)** LD_{50} of C-BLa 1.2 GpA_{+1} tested against other TMDs. **(b)** LD_{50} of C-BLa 1.2 GpA_{+1}G83I tested against other TMDs. **(c)** LD_{50} of C-BLa 1.1 LS46 tested against other TMDs. L16, LLLLLLLLLLLLLLLL; HLADQB, human leukocyte antigen β-subunit TMD, SGIGGFVLGLIFLGLGLII; LMP, TM5 of the Epstein-Barr virus latent membrane protein 1, LLAFFLAFFLDLILLIIALY); QSOX2, quiescin sulfhydryl oxidase 2 (see Fig. 1 b). TMDs in the N-BLa (N) or C-BLa (C) vectors are indicated below the x-axis. All LD_{50} values were normalized to the homodimerization signals (=100%) of GpA_{+1} (a, b) or LS46 (c). Ampicillin LD_{50} and GFP fluorescence values were measured in *E. coli* JM83 cells using the high-throughput assay protocol given in Supplementary Methods, with low-level induction (133 µM arabinose, 1 mM IPTG) for both BLaTM 1.1 and BLaTM 1.2 constructs. The GFP fluorescence was measured under equivalent conditions for all samples, adjusted by subtracting a cell-only control, and is expressed in fluorescence units per OD_{600}. Note that the fluorescence values given in (c) vastly exceed those in parts (a) and (b) which reflects higher protein expression associated with BLaTM 1.1.

Since the LD_{50} values are not raised in BLaTM 1.1, stronger expression is likely to lead to an accumulation of cytoplasmically localized protein; it follows that variations of fluorescence do not reflect varying levels of membrane-embedded proteins. Error bars show mean±SEM from at least 3 replicates.

Supplementary Methods

The high-throughput BLaTM assay was conducted using culture volumes of 300 µL in 96-well deep-well plates containing 2.2 mL wells (#701350, BRANDTECH) sealed with nonpermeable silicone lids (#3915707, VWR, Germany) reinforced with adhesive tape. Cultures were shaken at 700 rpm with an orbital diameter of 3 mm (Thermomixer HTM, HTA-BioTech, Bovenden, Germany). All LB media were supplemented with 0.125 M Na-phosphate pH 6.5 to increase ampicillin stability. Overnight culture medium was inoculated with 5 colonies using a pipette tip. After the overnight culture, cells were diluted 1:100 in expression medium. After the 4 hr expression culture, 16 µL of cells were added to 300 µL of fresh media containing the relevant ampicillin concentrations (calculated for a total volume of 316 µL), without adjusting cells to a fixed density. To measure the A_{600} and fluorescence levels, cells after the expression culture were washed twice in PBS (flow-cytometry grade, Gibco) by centrifugation of the plate at 2000 g for 10 min (5810R, Eppendorf). After resuspension to the original volume, 25 µL of cells were added to a black 96-well polystyrene microplate (237108, Nunc). The cells were diluted by adding 175 µL of PBS, and fluorescence measured (FLUOstar OPTIMA, λ_{Ex} = 485 nm, λ_{Em} = 520 nm, BMG Labtech). The cell density (OD_{600}) was measured using 150 µL of diluted cells in a clear microplate (VersaMAX, Molecular Devices), and adjusted to OD_{600} using a 0.3 cm path length. The GFP fluorescence per OD_{600} was adjusted by subtracting the value for a cell-only control (*E. coli* co-transformed with pACYC184 and pBAD322K, encoding chloramphenicol and kanamycin resistance, respectively). The OD_{600} after the LD_{50} culture was measured by transferring 150 µL to a clear microplate. The ECCpy module (see standard BlaTM protocol) was used for fully automated quality assessment of the sigmoidal curves, and the collection of validated data from multiple experiments. Quality benchmarks included an r^2 value > 0.9, a minimum of two datapoints above and below A_{600} 0.5, and a maximum standard deviation of OD_{600} 0.2 between datapoints at horizontal segments. All liquid transfer steps were conducted using multi-channel micropipettes (Eppendorf, Germany). All other conditions matched the standard BLaTM protocol.

Supplementary reference

1. Pelletier, J.N., Campbell-Valois, F.X. & Michnick, S.W. Oligomerization domain-directed reassembly of active dihydrofolate reductase from rationally designed fragments. *Proc Natl Acad Sci U S A* **95**, 12141-12146 (1998).
